# Hypermethylation of mitochondrial DNA facilitates bone metastasis of renal cell carcinoma

**DOI:** 10.7150/jca.62278

**Published:** 2022-01-01

**Authors:** Zheng Liu, Jinhai Tian, Fuhong Peng, Jiang Wang

**Affiliations:** 1 Department of Oncology, People's hospital of Dongxihu District, Wuhan, Hubei 430040, P.R.China.; 2 Department of Orthopedics, People's hospital of Dongxihu District, Wuhan, Hubei 430040, P.R.China.; 3 Department of Orthopedics, Tongji hospital of Tongji Medical College, Hua Zhong University of Science and Technology, Wuhan, Hubei 430030, P.R. China.

**Keywords:** Bone metastasis, clear cell carcinoma, mitochondria DNA, hypermethylation, 5-Azacytidine

## Abstract

Kidney cancers including clear cell carcinoma (RCC) are identified with very vulnerable mitochondria DNA (mtDNA) and frequent epigenetic aberrations. Bone metastasis from RCC is prevalent and destructive. Bone marrow contains a quite hypoxic microenvironment that usually insitigate 50% of hypermethylation events in conferring a selective advantage for tumor growth. We hypothesized that hypermethylation of mtDNA in RCC cells would significantly contribute to bone metastatic tumor progression. Methylation-specific polymerase chain reaction assay (MSP) was adopted to measure the methylation status of D-loop region of mtDNA in 15 pairs of bone metastatic and primary RCC as well as tumor adjescent normal kidney tissues. mtDNA copy number was examined by the real-time quantitative polymerase chain reaction (qPCR). Western blotting analysis was used to measure the accumulation of several DNA methyltransferases (DNMTs) in the mitochondria and nucleus fractions of bone metastatic RCC cells. mRNA expression of mitochondria encoded genes was examined by RT-PCR. Reactive oxygen species (ROS), mitochondrial membrane potential and ATP content were measured using *in vitro* cells treated with de-methylation drug 5-Azacytidine (5-Aza). Non-invasive bioluminescent imaging was performed to monitor tumor occurrence in skeleton in mice. Our results showed that the D-loop region in bone metastatic tumor cells was markedly hypermethylated than those in primary RCC tumor cells, that is associated with a decreased mtDNA copy number and accumulation of DNMT1 in the mitochondria. The bone-tropism tumor colonization and progression of RCC cells was significantly suppressed by demethylating the D-loop region of mtDNA and reducing the intracellular level of ROS and ATP by 5-Aza treatment. In conclusion, our study provided a direct association between hypermethylation of mtDNA in RCC with bone metastastic tumor growth.

## Introduction

Mitochondria are the crucial powerhouses of a cell 1. Mitochondrial dysfunction is a hallmark of cancer with commonly altered energy metabolism and has long-term been associated with metastatic tumor progression 2-4. Mitochondria also play important roles in regulating apoptosis and cell cycle, as well as maintaining cellular homeostasis in response to stresses, that are intrinsically critical to tumorigenesis 2, 3. The circular 16.6 kilobases mitochondrial genome DNA (mtDNA) encodes 13 essential proteins in the four respiratory chain complexes 5. The D-loop region in mtDNA is a unique 1124-bp non-coding region, controls mtDNA replication and mitochondrial gene transcription.

Kidney cancers are identified with very vulnerable mtDNA in the recent national cancer mitochondria genome project 6, for which the mtDNA structural variants and mtDNA gene expressions are highly altered among 38 common cancer types. This is partially due to the frequently activated hypoxia-inducible factor 1α (HIF1α) in this disease 4, and may also due to epigenetic reprogramming as kidney cancers are characterized with infrequent somatic mutations but frequent epigenetic aberrations 7.

Bone metastasis from kidney cancers especially clear cell carcinoma (RCC) is prevalent and particularly destructive 8. Approximately 50% of metastatic RCC patients have skeletal metastases with the 5 year survival rate of less than 10% 8. Bone marrow contains a distinctive hypoxic microenvironment 9. Evidence from multiple groups suggests that hypoxia or HIF1α signaling induces spontaneous metastasis to the bone and promotes tumor colonization and proliferation in the bone 10. Oxygen shortage not only activates hypoxia signaling pathways such as HIF1α, more profoundly, up to 50% of hypermethylation events in solid tumors are also due to hypoxia, and these events confer a selective advantage for tumor growth 11. We thus hypothesized that hypermethylation of mtDNA in RCC cells would significantly contribute to bone metastatic tumor progression. The direct impact and potential mechanisms (especially mtDNA mutations) of mtDNA on cancer cell behaviors including RCC have been extensively studied in recent years 12-16. Primary RCC tumors have a statistically lower mtDNA content than normal control subjects 17, and the resulting low mitochondrial respiratory chain components correlate with tumor aggressiveness 15. Although mutations of mtDNA had no significant impact on energy metabolism 14, they led to an irreversible shift to aerobic glycolysis in RCC 16. Other studies showed that D-loop region in colon cancer mtDNA was hypomethylated compared to the non-cancerous tissues 12, and the methylation status further decreased in advanced disease stages 12. De-methylation of CpG islands in the D-loop promoter of colorectal cancer can trigger cell proliferation, cell cycle progression and reduce apoptosis 12, which indicate that mtDNA methylation is negatively correlated with tumor progression. However, the understanding of mtDNA epigenetics has only recently gained recognition 18-20, the association and consequence of epigenetic regulation on mtDNA in bone metastasis from RCC has barely been investigated.

In supporting our hypothesis, we previously performed a proteomics analysis of bone metastatic RCC cells in comparing to the primary RCC cells, and identified altered *oxidative phosphorylation and mitochondria dysfunction* as the top-ranked signaling for the differential expressed intra-cellular proteins 21. In addition to oxidative phosphorylation, mitochondria are the major source of generating reactive oxygen species (ROS) in inducing oxidative stress which has been demonstrated in promoting cancer metastasis 22. Thus we further hypothesized that hypermethylation of mtDNA in RCC cells not only altered oxidative phosphorylation but also potentiated mitochondrial oxidative stress that actively promote bone metastasis.

Our current study provided evidence on a higher methylation rate at the D-loop region of mtDNA in bone metastatic than primary RCC tumor cells and patient tissues. We further demonstrated an accumulation of DNA methyltransferase (DNMT1) in the mitochondria of bone metastatic RCC cells. RNA analysis of mtDNA encoded genes showed a significant inverse between hypermethylation and decreased expression for many mitochondria genes. De-methylation with 5-Azacytidine (5-Aza), the inhibitor of DNMT, by significantly reducing the level of mtDNA methylation, decreased ROS production and intracellular ATP level of the bone metastatic RCC cells, thus prohibited bone metastatic tumor progression *in vivo*.

## Materials and methods

### Cell culture and compound

Human RCC cell lines ACHN and OS-RC-2, and normal human kidney cell line HEK293 were purchased from China Center of Type Culture Collection (CCTCC, Wuhan, China). Bone metastatic clones of the ACHN and OS-RC-2 cells, i.e., ACHN-BO and OS-RC-2-BO were established in our lab using the *in vivo* selection approach in nude mice 21, 23. All cells were maintained in DMEM/F12 (1:1) medium supplemented with 10% fetal bovine serum at 37°C in a humidified atmosphere of 5% CO_2_ in air. Primary normal human osteoblasts (HOB) cell pellet in RNAlater was purchased from Gene Company Ltd. (Beijing, China). 5-Aza (SelleckChem, Shanghai, China) stock solution in dimethyl sulfoxide (100 mmol/L) was stored at -20°C.

### Patient tissue samples

Fifteen sets of matched primary RCC tumor tissue and tumor adjacent healthy kidney tissue from nephrectomy and bone metastasis tissues from biopsy were retrospectively collected from tissue bank of Tongji Hospital. The clinical characteristics of the RCC patients are provided in Table S1. Inclusion criteria includes: patients with bone metastatic RCC histologically or cytologically documented, and adequate tissue for the molecular analysis; patients completed systemic therapy for advanced disease for at least 30 days. Patients with previous or concurrent malignancy, diabetes, or major medical illnesses were excluded. The diagnosis of primary RCC and bone metastasis as well as the corresponding matched tumor-free kidney tissue was done according to the WHO classification criteria by the Department Pathology of Tongji Hospital. The study was approved by the IRB committee of Tongji Medical College, Huazhong University of Science and Technology and was carried out in accordance with the Declaration of Helsinki. Consent forms from all participants were obtained.

### MSP assay

mtDNA was extracted from various cancer and normal cells and patient tissues using the Mito isolation kit (GenMed Scientifics, Shanghai, China). MSP analysis was conducted using the BisulFlash™ DNA Modification Kit (Epigentek, Shanghai, China) per the instruction.

### mtDNA copy number quantification

Real-time quantitative polymerase chain reaction (qPCR) was used to measure the relative mtDNA copy number. The primer pair for amplifying mtDNA are 5′-TACTCACCAGACGCCTCAACCG-3′ (forward) and 5′-TTATCGGAATGGGAGGTGATTC-3′ (reverse), for β-actin gene to standardize the input DNA are 5′-CGGGAAATCGTGCGTGACAT-3′ (reverse) and 5′-GAAGGAAGGCTGGAAGAGTG-3′ (reverse).

### Nucleus and mitochondrial fractionation

Cultured ACHN-BO and OS-RC-2-BO cells were treated with 5-Aza or control reagent for 24 hours, and then digested by 0.05% trypsin and resuspended in Mito-isolate buffer (20 mmol/L HEPES, 20 mmol/L KCl, 1.5 mmol/L MgCl_2_, 250 mmol/L sucrose, 1 mmol/L EDTA, 1 mmol/L PMSF, 1 mmol/L dithiothreitol, PH 7.5). The swelling cells were ruptured by glass homogenizer and then centrifuged at 700g for 10 minutes, and the sediment was preserved as nucleus fraction. The supernatant was centrifuged at 7000g for 10 minutes. The mitochondria sediment was washed by MS buffer (210 mmol/L mannitol, 70 mmol/L sucrose, 5 mmol/L Tris base, 5 mmol/L EDTA, PH 7.5). The nucleus and mitochondrial fraction were preserved for protein extraction. All experimentations were processed at 4°C.

### Western blot analysis

The nucleus and mitochondrial fractions were analyzed for protein expressions of DNMT1, DNMT3A, DNMT3B by Western Blot as we described previously 11, using GAPDH, nuclear marker Histone 3 and mitochondrial marker VDAC1 as controls. Primary antibodies against DNMT1 (Santa Cruz Biotech), DNMT3A (Abcam), DNMT3B (Abcam), GAPDH (Santa Cruz Biotech), VDAC1 (Abcam), and Histone 3 (Abcam) were used, donkey anti-rabbit/-mouse/-goat IgG (H&L) was used as second antibodies (Santa Cruz Biotech).

### RNA isolation and quantitative RT-PCR

RNA was extracted from cultured cells by TRIzol reagent (Life Technologies), and was reversely transcribed into complementary DNA by using Oligo (dT) primers. RT-PCR was performed using specific primer pairs (Table S2) with GAPDH gene as an internal control.

### Measurement of ROS and mitochondrial membrane potential

ROS levels in cultured cells were determined by using the DCFH-DA fluorescence indicator dye at a concentration of 10 μmol/L for 30 minutes in an incubator. Mitochondria membrane potential was determined by using the dye tetramethylrhodamine methyl ester (TMRM) at a concentration of 100 nmol/L for 30 minutes in an incubator. After incubation the cells were resuspended and washed by PBS and were then immediately monitored by flow cytometry.

### Measurement of ATP content

Total cellular ATP content was measured using the ATP Detection Kit (Beyotime) according to the manufacturer's instructions. In brief, the cultured cells were resuspended in lysis buffer, and the supernatant was added into 96-well plate with ATP detection buffer. The Relative Light Units (RLU) value was monitored by a luminometer and the ATP content was calculated using a standard curve.

### Seahorse extracellular flux assay

Experimental design for seahorse assay was on the basis of Agilent Seahorse XF Cell Mito Stress Test Kit (Agilent Technologies). ACHN-BO and OS-RC-2-BO cells were plated and grown on Agilent Seahorse XF Cell Culture Microplate in F12K media containing 10% FBS and then were treated with 5-Aza or control. Cells were then rinsed and cultured in XF Base Medium in 37°C incubator without CO_2_ for 1 hour. Traces of mitochondrial oxygen consumption rates (OCR) of the cells were measured with sequential injections of mitochondrial effectors (Oligomycin, FCCP, and Rotenone + antimycin A) at different time points. OCR was measured as the indicator of mitochondrial respiration.

### Animal experiments

6-8 weeks old female nude mice were purchased from the Experimental Animal Center of Huazhong University of Science and Technology (Wuhan, China). OS-RC-2-BO cells with or without 100nM 5-Aza pretreatment for 1 week (*in vitro*) were injected into mouse left cardiac ventricle at 5×10^5^ live cells in 0.1 ml PBS. Non-invasive bioluminescent imaging was performed to monitor tumor occurrence in skeleton twice per week for 8 weeks. All animal experiments were approved by the Animal Ethics Committee of Tongji Hospital affiliated Huazhong University of Science and Technology.

### Statistical analysis

Data are expressed as mean±SD from at least 2 independent experiments. Statistical analysis was done by GraphPad Prism 8.0 software. Parametric data with equal variance were analyzed by Student's t test. P<0.05 was considered with statistical significance.

## Results

### mtDNA D-loop region is hypermethylated in bone metastatic RCC cells and patient tissues

**Figure 1:**
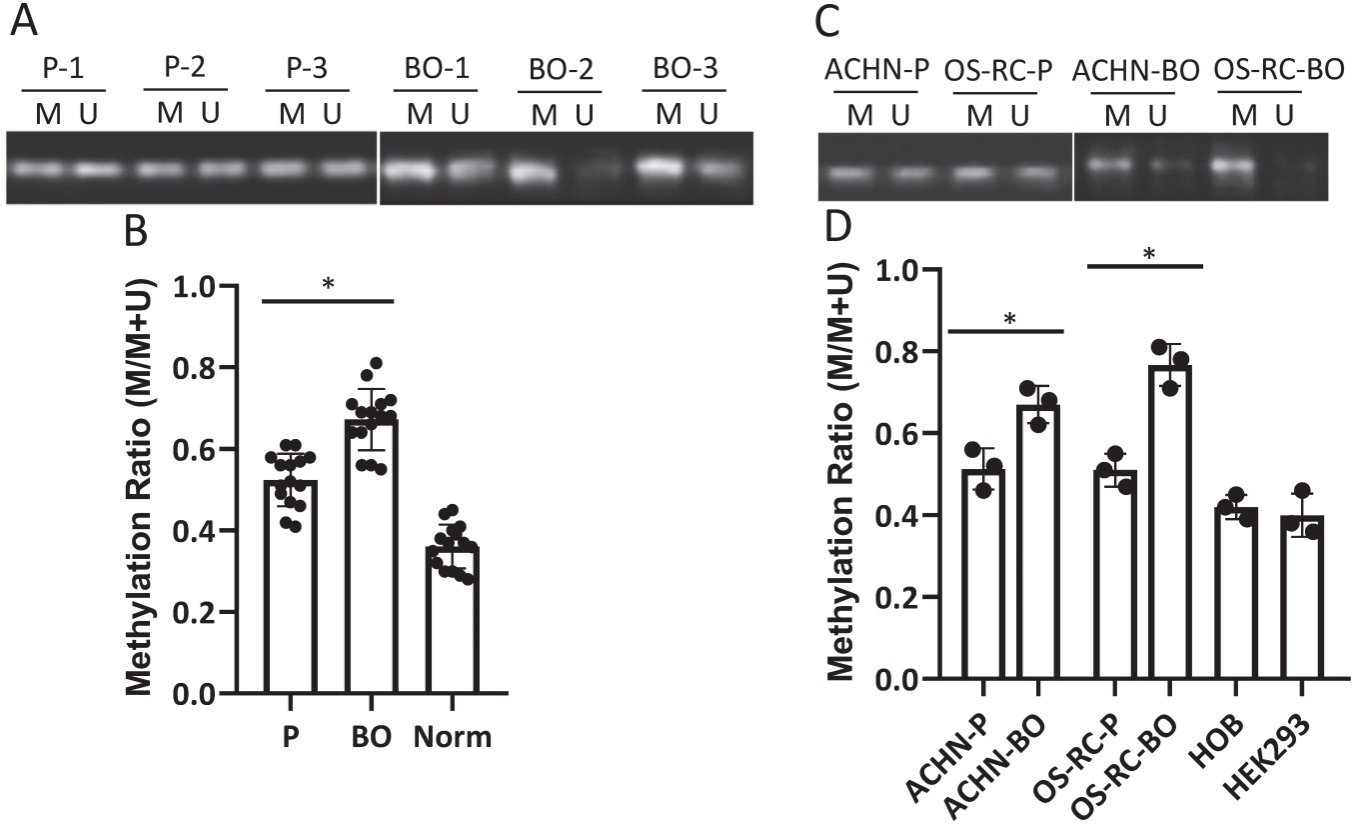
Methylation status of D-loop region in bone metastasis (BO) vs primary (P) RCC tumor and normal kidney tissue (Norm) specimens and cells. A. Representative MSP results of D-loop methylation in 3 pairs of BO and P tumor specimens. M: methylated, U: unmethylated. B. Statistical analysis of methylation ratios of D-loop region in all BO, P and Norm specimens (n=15). C. Methylation status of D-loop region in ACHN-P vs ACHN-BO and OS-RC-P vs OS-RC-BO cells. D. Statistical analysis of methylation ratios of D-loop region in tumor cell lines and normal human bone cell line (HOB) and human kidney cell line (HEK293) (n=3). *P < 0.05, by student's t-test. Error bars show ± SD.

D-loop region in mtDNA is a unique 1124-bp non-coding region, which controls mtDNA replication and mitochondrial gene transcription. The methylation rate of the D-loop region in the RCC patient cancerous and normal kidney tissue samples, the primary and bone metastatic RCC cell lines, the normal human kidney and bone cell lines were determined by the MSP assay. The results showed that the D-loop methylation rate in bone metastatic tumor tissues was markedly increased compared with that in primary RCC tumor tissues (52.3% in primary RCC tumors vs. 67.7% in bone metastatic tumors; P<0.05) (Fig. 1A, B). Strikingly, the bone metastatic ACHN-BO and OS-RC-2-BO cells are significantly hypermethylated in mtDNA D-loop in comparing to their counterpart parental cells (P<0.05) (Fig. 1C, D). In comparing to normal kidney tissues and human bone cells, the D-loop methylation of cancerous RCC cells are elevated (Fig. 1B, D) which is in accordance to previous findings 24.

### mtDNA copy number is decreased in bone metastatic RCC cells and patient tissues

**Figure 2:**
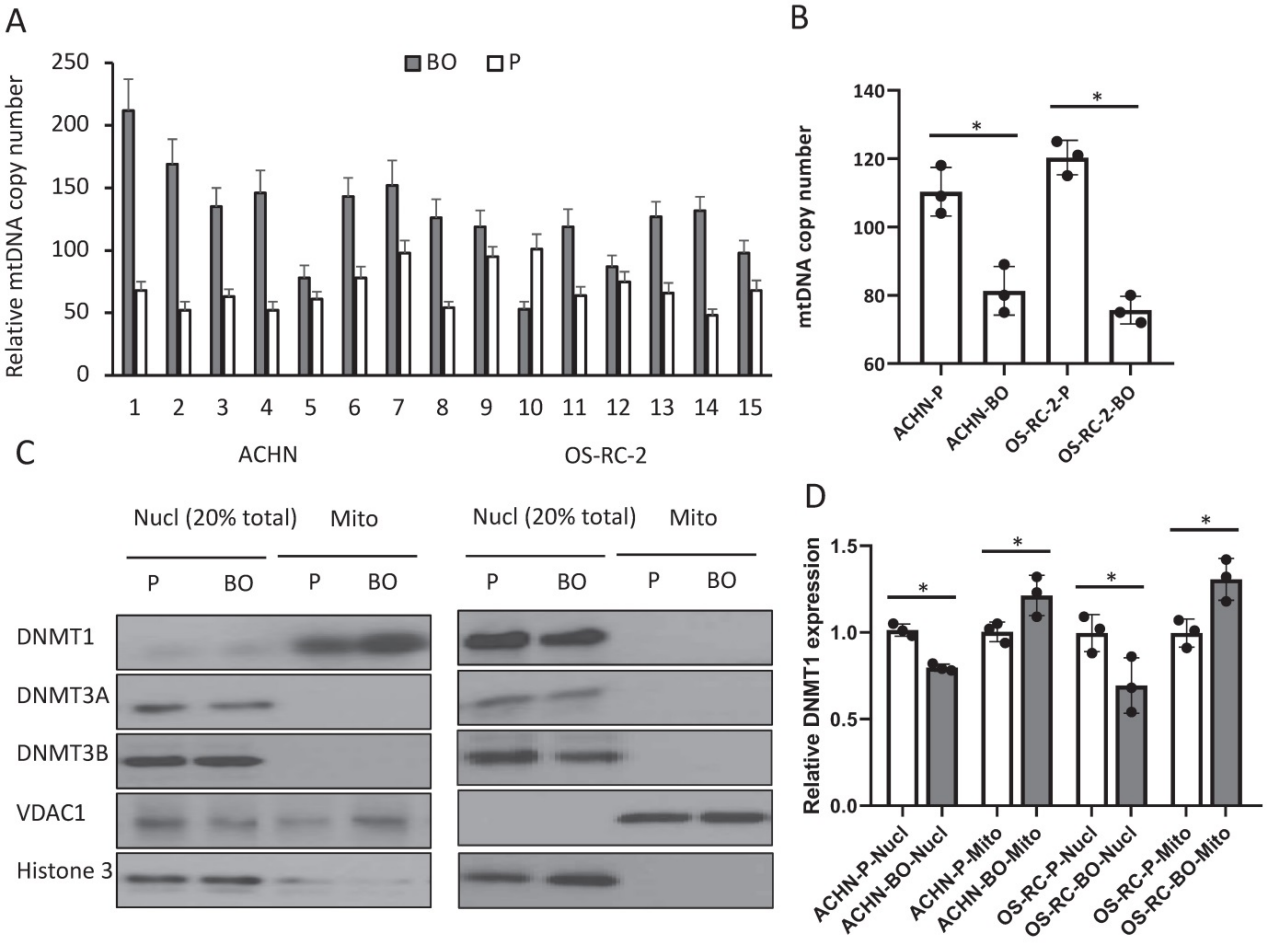
mtDNA copy number and mitochondria DNMT1 expressions in BO and P RCC tumor specimens and cells. A. Relative mtDNA copy number in 15 pairs of BO and P specimens. B. Comparison of the average relative mtDNA copy number between the 15 cases. * P<0.05. C. Representative blots of DNMT1, DNMT3A, and DNMT3B in nuclear (Nucl) and mitochondrial (Mito) fractions of ACHN and OS-CR-2 cells. D. Semi-quantification of DNMT1 and DNMT3A in C. Expression was normalized to the respective internal reference markers (VDAC1 or Histone 3), protein expression in P cells was set as 1. * P<0.05.

To examine whether the elevated D-loop methylation affect mtDNA duplication, we detected mtDNA copy number by qPCR. The relative mtDNA copy number was decreased in 13 (86.7%) bone metastatic tumor tissues when compared with that in primary RCC tumor tissues (126.4±38.5 in primary tumors vs. 66.7±18.7 in bone metastatic tumors; P<0.01) (Fig. 2A), as well as in the ACHN-BO and OS-RC-2-BO cells in comparing to the parental RCC cells (109±12% vs. 80±9% in ACHN-BO cells and 125±15% vs. 75±8% in OS-RC-2-BO cells) (Fig. 2B).

### Accumulation of DNMT1 in the mitochondria of bone metastatic RCC cells

To further explore the possible reason for the elevated D-loop methylation, we examined the expressions of several DNMTs that catalyze the transfer of a methyl group to DNA. Immunoblots of nuclear and mitochondrial fractions from cultured RCC cells showed both DNMT1 and DNMT3A expressions (Fig. 2C), while DNMT3B only exists in the nuclear (Fig. 2C). ACHN-BO and OS-RC-2-BO cells did not show an altered total DNMT1 expression but had an increased mitochondrial accumulation of DNMT1 (Fig. 2C, D). These results suggested that hypermethylation of the mtDNA in bone metastatic tumor cells may likely due to the accumulated DNMT1 in mitochondria.

### Hypermethylation of mtDNA is associated with decreased mitochondria gene expression

**Figure 3:**
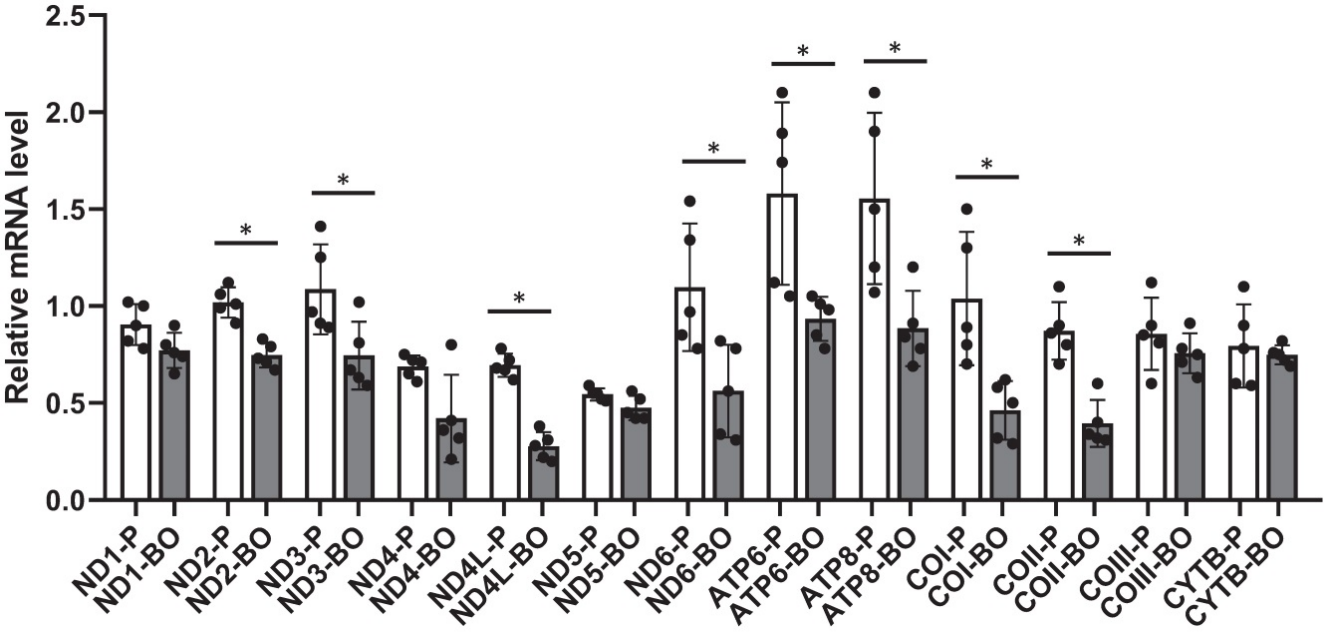
The mRNA expression levels of mtDNA genes in BO vs P RCC cells detected by quantitative RT-PCR (n = 5). * P<0.05.

To further confirm the negative regulation of hypermethylation on gene transcription, transcriptions of mitochondrial exclusive genes were analyzed by RT-PCR. Results showed that mRNAs encoding NADH dehydrogenase 2 (ND2), ND3, ND4L, ND6, ATP synthase 6 (ATP6), ATP8, cytochrome c oxidase I (COI), and COII were down-regulated in the ACHN-BO and OS-RC-2-BO cells in comparing to those in the parental ACHN and OS-RC-2 cells (Fig. 3).

### De-methylation with 5-Aza suppressed bone metastatic tumor progression

**Figure 4:**
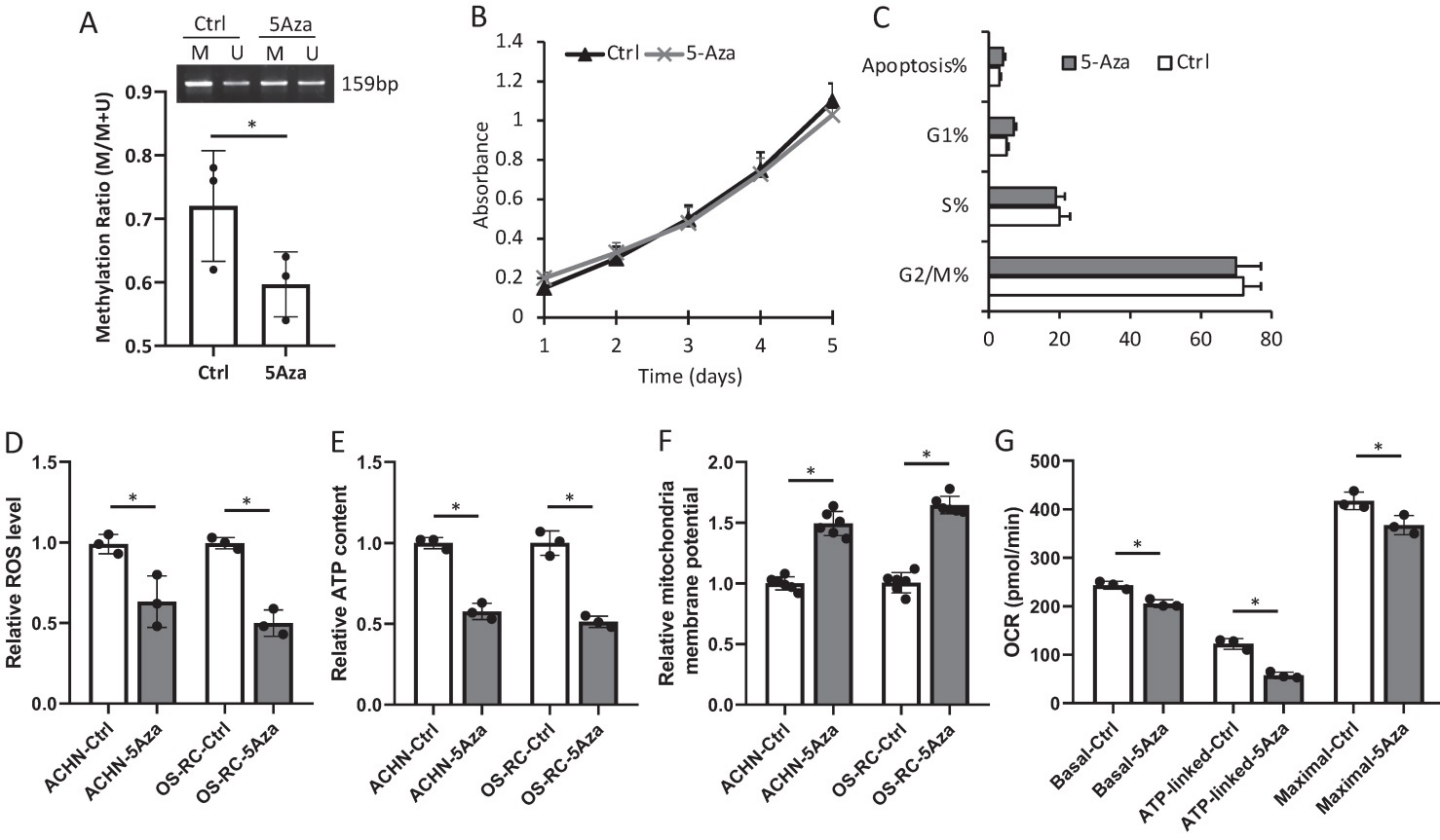
*In vitro* effects of 5-Aza on D-loop methylation (A), cell proliferation (B) and apoptosis (C), as well as mitochondria function (D-G) in ACHN-BO and OS-RC-2-BO cells. *P < 0.05 by student's *t-*test. Error bars show ± SD.

Treatment with 100nM 5-Aza for 1 week was confirmed to alter the D-loop region from a methylation to a de-methylation status in OS-RC-2-BO cells, but had very minimum effects on *in vitro* cell proliferation and apoptosis (Fig. 4A-C). We further found that 5-Aza treatment decreased the ROS production and intracellular ATP level, and increased mitochondrial membrane potential of the ACHN-BO and OS-RC-2-BO cells (Fig. 4D-F). As measured by the seahorse extracellular flux analyzer, the 5-Aza treatment significantly suppressed basal, ATP-linked, and maximal OCR in OS-RC-2-BO cells (Fig. 4G).

**Figure 5:**
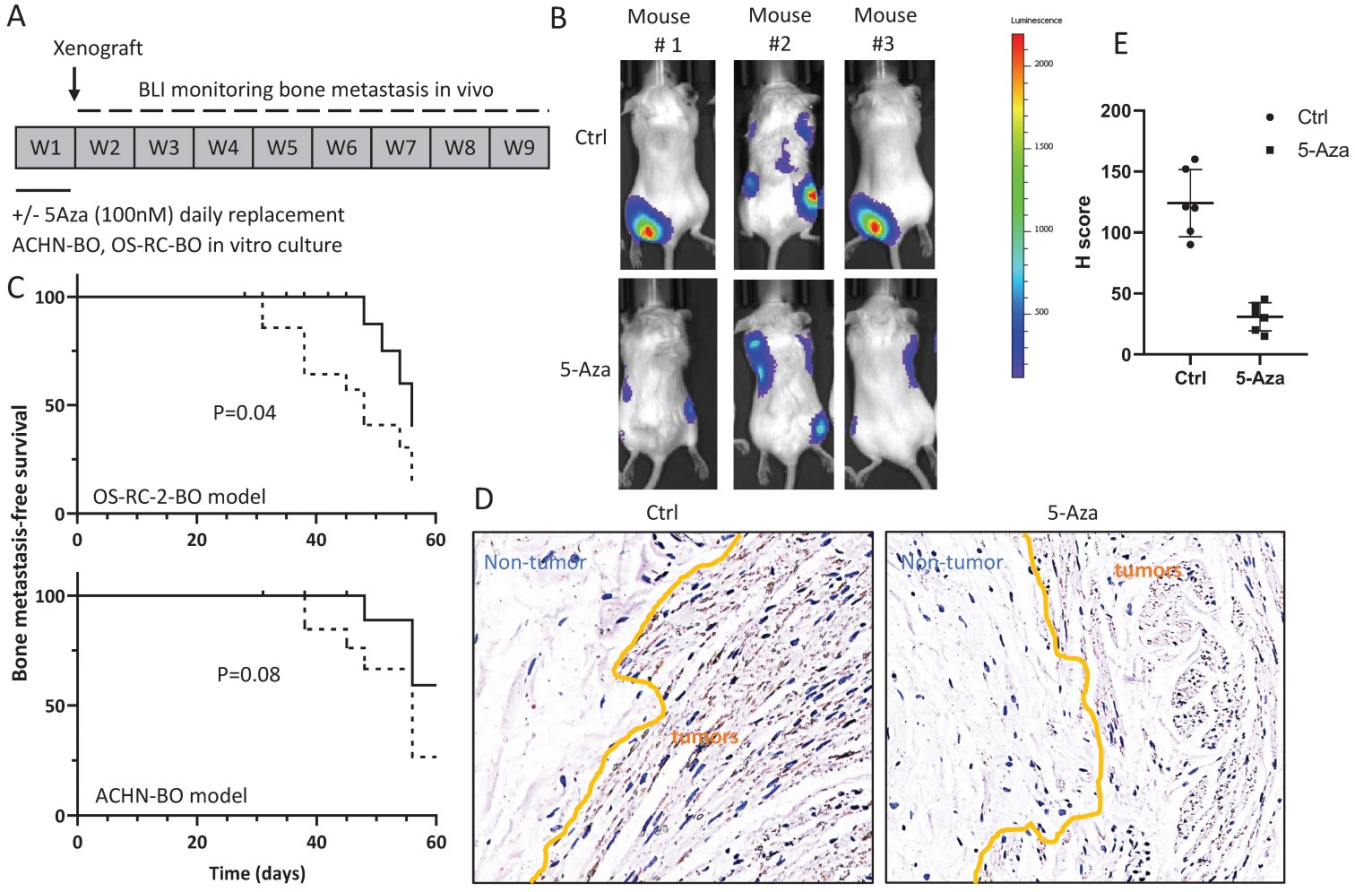
Treatment with 5-Aza on bone metastatic tumor development in xenograft animal models. A. Experimental design for the assessment of *in vivo* bone metastatic tumor growth after epigenetic methylation reprogramming. ACHN-BO and OS-RC-2-BO cells were pretreated with 100nM 5-AZA for 1 week *in vitro*. The reprogrammed and control cells were injected into the left cardiac ventricle of athymic nude mice. Tumor growth in skeleton (spine or limbs) were monitored twice a week for 8 weeks. B. Representative bioluminescent images of bone metastasis in 3 mice in the control and 5-Aza treatment groups. C. Kaplan-Meier curves for the bone metastasis-free survival of both ACHN-BO and OS-RC-2-BO xenograft animal models. The *p* value was calculated with a *log-rank test* (*N* = 14). D. Representative images of HIF1α immunohistochemistry staining and quantification (H scores) of the HIF1α immuno-reactivities in the bone tissue sections from the mice who developed bone metastasis. Images were taken under 10x objective.

In mice, the 5-Aza pretreated ACHN-BO and OS-RC-2-BO cells showed remarkable deficiency in inducing skeleton metastasis when injected through left cardiac ventricle (Fig. 5A, B). In specific, in the OS-RC-2-BO model, 71% (10/14) mice developed metastasis in spine or limbs by the end of 8 weeks, while only 28.5% (4/14) mice in the 5-Aza pretreatment group were examined with obvious metastasis in hind legs (P=0.04) (Fig. 5C). Similarly, 5-Aza prohibited ACHN-BO cells to metastasize to skeleton from 50% to 20% of mice (P=0.08) (Fig. 5C). We further examined the HIF1α expression by immunohistochemistry staining of the bone tissue sections from the mice who developed bone metastasis, and quantified the results using H score, which was calculated by multiplying the fraction of positively stained tumor in the whole section image (percentage) by staining intensity (0, 1+, 2+, or 3 +). The data shown that bone metastatic tumors in the 5-Aza treat mice had a significantly reduced HIF1α immune-reactivity (P<0.01) (Fig. 5D).

## Discussion

mtDNA encodes a limited number of protein that is usually regarded as a less important player in cancer development. Emerging studies identified expanded roles of mtDNA in impacting tumorigenesis and metastatic progression 25, 26. Our study provided a direct association between hypermethylation of mtDNA in RCC with bone metastastic tumor growth at the first time. We further demonstrarted that the bone-tropism tumor colonization and progression of RCC cells could be significantly suppressed by demethylating the D-loop region of mtDNA and reducing the intracellular level of ROS and ATP by 5-Aza treatment.

One key question is what and how signals from mitochondria coordinately regulate the large number of molecules involved in the metastatic cascade? Cues from early mitochondrial evolution suggest that retrograde (from mitochondria to nucleus) signals are coupled with anterograde (from nucleus to mitochondria) signals to determine how cells respond to stress in regaining cellular homeostasis 27. One critical component in this response process is regulation of protein secretion and placement in organelles 28. In our previous studies, we identified that the bone-metastatic RCC cells were able to secrete significantly more stress-induced phosphoprotein 1 (STIP1) protein than the primary RCC cells 29, not only promoted the proliferation and migration/invasion of RCC tumor cells through the autocrine receptor, but also facilitated the differentiation of osteoclasts and up-regulated cathepsin K through paracrine, to degrade collagen and other matrix proteins during bone resorption 29. Multiple mechanisms used by mitochondria to transduce signal to nucleus include ROS, tricarboxylic acid cycle metabolite, calcium homeostasis, and AMPK activation 30, 31, which are also critical in controlling metastasis. In the current study, we examined an over-production of ROS in the bone-metastatic RCC cells. Extensive evidence has shown that ROS promotes many aspects of tumor development and metastasis 32. A recent study reported that the STIP1 homology Stub1 is necessary for protective degradation of ROS-stressed peroxisomes 33, we thus postulate that the ROS-STIP1 signaling, in maintaining a delicated balance of intracellular ROS level, is critical for RCC cells to detoxify from ROS and contributes to bone metastasis. In addition, Koshikawa et al. demonstrated that increased mitochondria ROS resulted in increased transcription of metastasis-associated genes 34, 35. Our study doesn't exclude this possibility, but it would be interesting to explore the relevance of increased ROS in bone-tropism metastasis in RCC.

We are aware that the patient samples used for the mtDNA mythelation analysis are very limit. However, this would not compromise the novelty and scientific conclusion of the study, first, similar studies in RCC bone metastasis has never been proposed before, second, the paired samples from each patient including primary RCC and bone metastasis as well as normal kidney tissue are kind of unique. Nevertheless, we tried to mine public available clinical cohorts, unfortunately, most genome-wide association studies (GWAS) or epigenome-wide association studies (EWAS) studies did not include mtDNA in genomic analyses. Technically, sequencing tumor mtDNA is also challenging as it's easily get contaminated with normal tissue and there is a lack of definitive detection due to multiple copies and heteroplasmy 36. Therefore, in the current study, we chose to examine the overall methylation of the mtDNA D-loop region by the methylation-specific PCR assay. This PCR-based assay is extremely sensitive, facilitating the detection of low numbers of methylated alleles in samples containing small amounts of DNA such as mtDNA. Although this assay does not provide absolute quantification of DNA methylation at the single base-resolution, our study design was well justified in considering the limited sample size and large numbers of single-base methylation events in previous reports 24, 37. Despite these, with more recognizing the importance of mtDNA, more mtDNA-included genomic studies will be performed to improve our understanding of mtDNA in tumor progression and metastasis suceptability.

## Supplementary Material

Supplementary tables.
