# Supplementary material for: Hypermethylation of mitochondrial DNA facilitates bone metastasis of renal cell carcinoma
Source: J Cancer. 2022 Jan 1;13(1):304–12. doi: 10.7150/jca.62278 (PMC8692697; doi:10.7150/jca.62278)
Supplement: Supplementary file 1 — Supplementary tables. [file jcav13p0304s1.pdf]

Table S1. Clinical and pathologic features of the RCC cohort

ID	Age at surgery	Gender	Differentiation stage	Pathologic T	N stage	Tumor size (Max Dimension)	Nephrectomy	Biopsy location of BM
C1	59	Female	G2	pT2b	N0	101 mm	radical, right	Sacrum
C2	47	Male	G2	pT3a	N1	71 mm	radical, right	Sacrum
C3	62	Male	G3	pT1b	N1	65 mm	radical, left	Long bones
C4	60	Male	G2	pT1a	N1	28 mm	radical, left	Spinal column
C5	61	Female	G2	pT3a	N1	75 mm	radical, left	Spinal column
C6	68	Male	G3	pT2b	N2	60 mm	partial, right	Sacrum
C7	58	Female	G1	pT3a	N1	98 mm	radical, right	Long bones
C8	46	Female	G2	pT3b	N1	102 mm	radical, right	Sacrum
C9	49	Female	G2	pT21	N1	64 mm	radical, left	Spinal column
C10	62	Male	G2	pT3a	N0	84 mm	radical, right	Sacrum
C11	57	Male	G3	pT2a	N1	85 mm	radical, right	Spinal column
C12	48	Female	G2	pT2b	N2	62 mm	radical, left	Sacrum
C13	68	Female	G2	pT1b	N2	55 mm	radical, left	Long bones
C14	53	Male	G3	pT3b	N1	58 mm	radical, right	Spinal column
C15	60	Male	G3	pT2a	N1	72 mm	radical, left	Sacrum

Table S2. Primer sets for RT-PCR

RT-PCR Primers for mt genes	Sequence
ND1	5’-CGATTCCGCTACGACCAACT-3’
	5’-AGGTTTGAGGGGGAATGCTG-3’
ND2	5’-ACCAAACCCAGCTACGCAAA-3’
	5’-AGTAGTAGGGTCGTGGTGCT-3’
ND3	5’-GCGGCTTCGACCCTATATCC-3’
	5’-AGGGCTCATGGTAGGGGTAA-3’
ND4	5’-TCGCTCACACCTCATATCCTC-3’
	5’-AGGCGGCAAAGACTAGTATGG-3’
ND4L	5’-CAGCCACATAGCCCTCGTAG-3’
	5’-CCCGTGGGCGATTATGAGAA-3’
ND5	5’-TCATCGCTACCTCCCTGACA-3’
	5’-ATCCTGCGAATAGGCTTCCG-3’
ND6	5’-ACCTATTCCCCCGAGCAATC-3’
	5’-GGGAGGATCCTATTGGTGCG-3’
ATP6	5’-CGTACGCCTAACCGCTAACA-3’
	5’-AGGCGACAGCGATTTCTAGG-3’
ATP8	5’-TACCACCTACCTCCCTCACC-3’
	5’-AGGATTGTGGGGGCAATGAAT-3’
COI	5’-ACCCTAGACCAAACCTACGCCAAA-3’
	5’-TAGGCCGAGAAAGTGTTGTGGGAA-3’
COII	5’-CCGTCTGAACTATCCTGCCC-3’
	5’-GAGGGATCGTTGACCTCGTC-3’
COIII	5’-CAGCCCATGACCCCTAACAG-3’
	5’-TACATCGCGCCATCATTGGT-3’
Cytb	5’-AGTCCCACCCTCACACGATTCTTT-3’
	5’-AGTAAGCCGAGGGCGTCTTTGATT-3’
GAPDH	5'-AATGGGCAGCCGTTAGGAAA-3'
	5'-GCCCAATACGACCAAATCAGAG-3
